# Fiction is Sweet. The Impact of Media Consumption on the Development of Children’s Nutritional Knowledge and the Moderating Role of Parental Food-Related Mediation. A Longitudinal Study

**DOI:** 10.3390/nu12051478

**Published:** 2020-05-19

**Authors:** Alice Binder, Brigitte Naderer, Jörg Matthes, Ines Spielvogel

**Affiliations:** 1 Advertising and Media Effects Research Group, Department of Communication, University of Vienna, 1090 Vienna, Austria; joerg.matthes@univie.ac.at (J.M.); ines.spielvogel@univie.ac.at (I.S.); 2 Department of Media and Communication, Ludwig-Maximilians-University Munich, 80538 Munich, Germany; Brigitte.Naderer@ifkw.lmu.de

**Keywords:** nutritional knowledge, media, children, parental food-related mediation styles, individual preconditions

## Abstract

Nutritional knowledge is an important cognitive facilitator that potentially helps children to follow a healthy diet. Two main information agents influence children’s development of nutritional knowledge: the media and their parents. While a high amount of media consumption potentially decreases children’s nutritional knowledge, parents may shape the amount of information children can gather about nutrition through their food-related mediation styles. In addition, children’s individual preconditions predict how children can process the provided nutritional information. This two-wave panel study with children (*N* = 719; 5–11 years) and their parents (*N* = 719) investigated the main effects and interplay of children’s amount of media consumption and their parents’ food-related mediation styles by performing linear regression analysis. Children’s individual preconditions were also considered. We measured children’s self-reported amount of media consumption, children’s age, sex, weight, and height (BMI). Additionally, in a parent survey we asked parents about how they communicate their rules about eating while especially focusing on active and restrictive food rule communication styles. As a dependent measure, we examined children’s nutritional knowledge at Time 1 and 2. The results show that the amount of media consumption has a negative effect on children’s nutritional knowledge over time. Parents’ restrictive or active food-related mediation asserted no main effects and could not lever out the negative effect of the amount of media consumption. Therefore, we argue that parents should limit children’s amount of media consumption to avoid the manifestation of misperceptions about nutrition.

## 1. Introduction

Healthy ingredients such as milk and honey are mixed together and suddenly a tasty milky bar surfaces; fruit gums appear in connection with the word ‘vitamins’ while fresh fruit is also visually presented. These are just two examples of recent commercials of products high in added sugar, targeting children and their parents. These commercials try to convince the audience that the promoted products have a higher nutritional value than is actually the case. Hence, the presented commercials spread misleading or wrong information about products, which can potentially distort children’s and parents’ perceptions of what “healthy” actually means and, thus, negatively impact their nutritional knowledge.

Misleading health claims in connection with unhealthy foods are heavily used within audiovisual commercials [1]( see for a review [2]) and editorial content alike (e.g., TV shows, movies) [3]. Furthermore, unhealthy food presentations dominate the media environment in general [4,5,6,7,8]. Effect studies indicated that unhealthy food presentations within media content can influence children’s attitudes [9], intentions [10], and actual behavior [11]. The overrepresentation of unhealthy foods and the presence of misleading health claims within media content may lead to a subsequent decrease in children’s overall health, e.g., through an increase in their Body Mass Index (BMI) [12]. This is problematic because high levels of BMI are frequently connected to health risks such as cardiovascular diseases, diabetes, musculoskeletal disorders, and some forms of cancer. Children who are obese or overweight in a younger age are furthermore more likely to be overweight or obese when they are adults [13], which is associated with additional, long-lasting health problems [14,15].

Nutritional knowledge might be one strategy to partly shelter children from these negative effects [16]. That is, children might thus need cognitive control in order to choose more healthy foods over unhealthy foods and to overcome their instinct of eating unhealthy over healthy food options [17]. This cognitive control can be achieved through nutritional knowledge [18].

In our study, we focus on procedural nutritional knowledge which describes knowledge about how to correctly evaluate which foods contain which nutritional ingredients [19,20]. With regard to media and advertising effects on the development of nutritional knowledge, studies already showed that health claims on food packaging such as showing fruit or describing a product as low in fat let children between the age of 5 to 10 years conclude that these products were healthy independent of their true nutritional value [21]. Along those lines, studies have indicated that misleading health claims are influencing the evaluation of the nutritional value of unhealthy foods [22,23,24,25]. However, we lack insights into what happens to children’s development of nutritional knowledge if they are confronted with an accumulation of misinformation about nutrition, typically found in TV commercials and other media presentations [18]. We also lack non-experimental studies, given that experiments are limited in their generalizability. In a pioneering study with 172 children, Harrison [18] examined the effects of television viewing on children’s nutritional knowledge over a period of six weeks. The author presented two food pairs and asked the children which one of these foods can be categorized as healthier than the other one. It was found that children’s television viewing frequency negatively impacted children’s nutritional knowledge. Hence, it can be assumed that, through misinformation about nutrition on TV, children’s nutritional knowledge may deteriorate. Yet, Harrison [18] found this effect only for diet foods (e.g., fat-free ice cream) but not for non-diet foods. However, the author suggested that a bigger sample and a longer time period between the two waves would be essential to adequately assess the impact of media consumption on the development of children’s nutritional knowledge.

Additionally, the amount of media consumption is only one aspect that is responsible for children’s development of nutritional knowledge. The family context is a highly important factor for developing children’s basic understanding of what they know about food, and what kind of food they like [26,27]. Especially, parents’ communication styles are particularly important. This is because parents’ communication about food, also referred to as food-related mediation, can be either focused on explaining and reasoning nutritional decisions providing children with context and information (i.e., active food-related mediation), or their communication can only be based on enforcing restrictions and rules (i.e., restrictive food-related mediation) [28]. While a restrictive mediation style involves communicating prohibitions and rules but not rationalizing them for children, active mediation involves not only rationalizing rules but also articulating possible consequences of violating them [28,29]. Only two studies we know of have examined restrictive and active mediation styles with regard to parental communication about food [11,30]. Yet, we cannot generalize these findings to cognitive concepts such as nutritional knowledge. In addition, no study we know of has examined media and parental influences at the same time. Furthermore, how children learn and internalize nutritional knowledge might be highly dependent on their individual preconditions. Hence, with our study, we further contextualize the development of children’s nutritional knowledge by considering children’s BMI, age, and sex [31].

**Figure 1:**
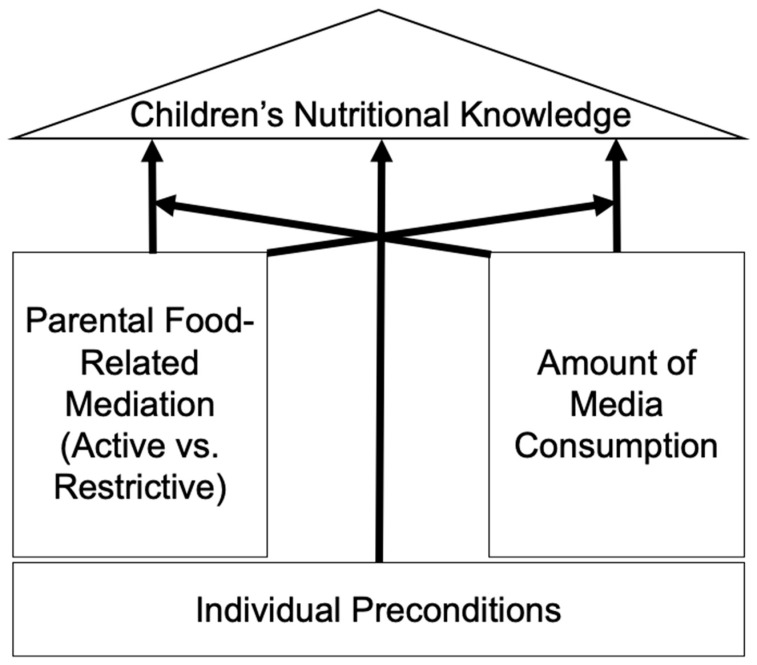
Conceptual model.

We therefore build on the conceptualization of children’s nutritional knowledge development that asserts for (A) individual preconditions of the children, (B) their interpersonal environment, and (C) their media environment—as agents for knowledge. We consider the latter two agents for knowledge based on Bandura (1969), who assumed that children do not only learn from their interpersonal environment but also from mediated experiences. As these two agents of knowledge could potentially provide diverging information, we furthermore consider their interaction effect (see Figure 1 for a visualization of the conceptual model). In the present study, we measure children’s nutritional knowledge with a focus on knowing about the ingredients of food as this seems very important for the examined age group of children between 5 and 11 years old [21].

## 2. Materials and Methods

We collected the data from children and one of their parents in 12 primary schools (102 classes) in rural (50%) and urban (50%) areas in Austria based on convenience sampling. Overall in Austria, the number of overweight and obese children is increasing. This is especially true for children in the tested parts of Austria in which 32.3% of the boys and 29.1% of the girls in the 3rd grade can be categorized as overweight or obese [32]. The first wave was conducted between 21 March and 23 May in 2019. The second wave was implemented six months after the first wave between the 4 September and 28 November 2019.

The headmasters of the participating schools and the ethical committee of the University of Vienna (Ethics Approval Number: 00343) approved the study. For each child, we obtained his or her oral consent to participate in the study as well as his or her parents’ written consent. We informed children as well as their parents that they could withdraw their consent or withhold information at any point of the study.

Two researchers interviewed the children individually in both waves in separate interview rooms in the participating school. Additionally, after the interview, we measured children’s weight and height to assess the BMI. To measure parental food-related mediation styles, we matched the data of each child with the survey completed by his/her parent. The same procedure was followed in the second wave of the study.

### 2.1. Participants

Our final sample included 719 children (*M_age_* = 7.80, *SD* = 1.23; age range 5–11 years; 46.2% female (*N_missing_* = 4)) and one of their parents (*M_age_* = 40.02, *SD* = 5.74 (*N_missings_* = 34); 79.5% female (*N_missings_* = 15)). This final sample represents those children who participated in both waves and could be matched to their parents’ questionnaires at Time 1. Approximately 2250 documents in 12 schools with 102 school classes were initially distributed. Overall, 829 parents returned documents back to the schools (response rate: 36.84%). At Time 1, we had 795 children participating in the study whereas at Time 2, 734 of these children were interviewed again. Thus, 92.33% of the children participated in both waves. The sample for this study consists of 719 children who participated in both waves and responses of their parent (*N_missings_* = 110 from the initial sample of *N* = 829 due to missing data from parents and/or children).

### 2.2. Measures

This study was part of a larger project. Only measures relevant to the present study are described in detail below.

*Independent variables.* Because there is a trend that children have their own TV and computer in their rooms [33], and based on other studies in this area of research [18], we asked children directly about their amount of media consumption, not their parents. We asked two separate questions: How often children are allowed to watch TV series and how often they are allowed to watch movies on a 4-point Likert scale (1 = never; 4 = as long as I want; Cronbach’s α_(*t*1)_ = 0.63; *r*(705) = 0.46, *p* < 0.001; *M*_(*t*1)_ = 2.57, *SD*_(*t*1)_ = 0.65; *N_missings_* = 5).

We asked parents six items about food-related mediation styles, each rated on a 7-point Likert scale (1 = seldom; 7 = very often). The items are based on the established media mediation measurements of active and restrictive mediation strategies [11]. The six items were simultaneously entered into an explorative principal component analysis with oblique rotation. The analyses yielded two factors, explaining 74.74% of the variance. Three items represented restrictive food-related mediation, e.g., “I follow clear rules and restrictions on what my child is allowed to eat” (Cronbach’s α_(*t*1)_ = 0.77; *M*_(*t*1)_ = 4.03, *SD*_(*t*1)_ = 1.73; *N_missings_* = 6). Three other items represented active food-related mediation, e.g., “I talk to my child about why a food is healthy or unhealthy” (Cronbach’s α_(*t*1)_ = 0.87; *M*_(*t*1)_ = 5.62, *SD*_(*t*1)_ = 1.43; *N_missings_* = 9). Both measures were reliable (see Appendix A).

To collect data for the children’s age, we simply asked each child how old he or she was at the time of the interview (*M*_(*t*1)_ = 7.80, *SD*_(*t*1)_ = 1.40).

For children’s BMI, we measured children’s height and weight and the standard deviation score of BMI (zBMI) was computed to adjust for children’s age and sex (*M*_(*t*1)_ = 0.15, *SD*_(*t*1)_ = 1.09; *N_missings_* = 8) [34]. The results show that 21.7% (*n* = 156) of all children in this study had a BMI score above the cut-offs of normal weight and are thus characterized as overweight (+1SD; 18.3% of the sample) or obese (+2SD; 3.4% of the sample) [13].

*Dependent variable.* Our dependent variable was measured in both waves to assess the autoregressive effect over time. Since studies already showed that in some countries asking children which of two foods is healthier is too easy for children [35] and this might be especially true in an European context, we used a more detailed measurement. Adapted from Tarabashkina and colleagues [35], we assessed children’s nutritional knowledge using three illustrated questions. These questions measured children’s knowledge about foods that had either high or low content of fat, salt, and sugar. Since nutritional claims are frequently referring to these ingredients [3], this measurement seems appropriate. Moreover, with these items we are able to measure children’s procedural nutritional knowledge [19]. Children had four answer options to three different questions (e.g., “Which one of the presented foods has the lowest amount of fat?”). There was always only one correct answer. We later dummy-coded children’s answers (1 = correct answer; 0 = incorrect answers) to calculate their nutritional knowledge and summed up the three dummies resulting in a summated index ranging from 0 to 3. While the questions themselves stayed unchanged between the two waves, the pictorial food options were updated to prevent possible memory effects. However, we tried to pick different items within the same food category (e.g., answer option Time 1: Cola; answer option Time 2: Sprite; *M*_(*t*1)_ = 1.85, *SD*_(*t*1)_ = 0.87; *N_missings_* = 10; *M*_(*t*2)_ = 1.92, *SD*_(*t*2)_ = 0.89; *N_missings_* = 11). For details on the measurement see Appendix A.

### 2.3. Data Analysis

We calculated a linear regression analysis in SPSS [36] predicting Time 2 of children’s nutritional knowledge while assessing children’s nutritional knowledge of Time 1 (*b* = 0.32, β = 0.32, *p* < 0.001). In a first step, we calculated the effects of the individual preconditions such as children’s age, BMI, and sex at Time 1 as well as nutritional knowledge at Time 1. In the next step, we calculated the main effects of children’s amount of media consumption and parental mediation styles. In a third step, we calculated interaction effects of active or restrictive parental food-related mediation strategies and children’s amount of media consumption.

## 3. Results

### 3.1. Amount of Media Consumption

Table 1 displays the results of the linear regression analysis predicting children’s nutritional knowledge at Time 2. Children’s amount of media consumption offered a significant negative prediction for children’s nutritional knowledge (*b* = −0.14, β = −0.12, *p* = 0.003). Thus, the higher children’s amount of media consumption at Time 1 the lower was children’s nutritional knowledge at Time 2.

### 3.2. Parental Food-Related Mediation Strategies

**Figure 2:**
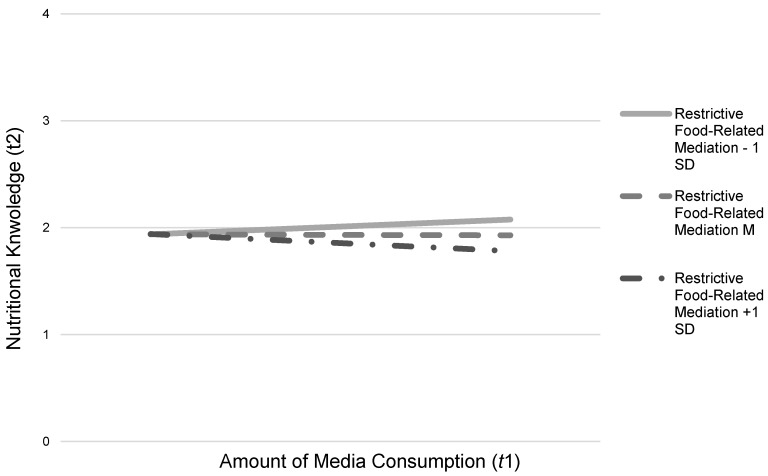
Interaction effects of children’s amount of media consumption (*t*1) and restrictive food-related mediation (*t*1) on children’s nutritional knowledge (*t*2).

The results showed no main effects of parents´ active food-related mediation style (*b* = 0.03, β = 0.05, *p* = 0.232) or restrictive food-related mediation style (*b* = −0.01, β = −0.02, *p* = 0.726) on children’s nutritional knowledge. Additionally, the interaction between children’s media consumption and parents’ active food-related mediation style did not indicate any effects on children’s development of nutritional knowledge (*b* = −0.01, β = −0.01, *p* = 0.866). However, we found a significant interaction effect of children’s amount of media consumption and parents’ restrictive food-related mediation style on children’s nutritional knowledge (*b* = −0.07, β = −0.10, *p* = 0.017). The nature of this effect is displayed in Figure 2. The effect showed that media consumption on nutritional knowledge was negative if the parental food-related mediation was high. Additionally, we also examined the Johnson-Newman table to investigate at what level of a restrictive food-related mediation this negative interaction effect occurs [36]. The results showed that, on an average to high level of restrictive food-related mediation (above 3.40 on a 7-point scale), the effect of children’s media consumption on nutritional knowledge is significantly negative. For low restrictive mediation, the results showed no significant effects (under 3.39).

### 3.3. Individual Preconditions

The results indicate that children’s age is a positive predictor of children’s nutritional knowledge (*b* = 0.13, β = 0.18, *p* < 0.01). For children’s zBMI (*b* = −0.01, β = −0.01, *p* = 0.726) or children’s sex (*b* = 0.01, β = 0.01, *p* = 0.870), we did not find significant effects.

## 4. Discussion

The present research provides important insights into how both the amount of media consumption as well as parental food-related mediation are influencing children’s nutritional knowledge over time. Our study is unique because we employed an externally valid design using a large sample size of children, combining survey data of children with the data of their parents, controlling for the autoregressive effect of nutritional knowledge, and using a timespan. There are three main findings. First, our data suggest that media consumption negatively predicts the development of children’s nutritional knowledge. In other words, consuming a high number of movies and TV-series shaped children’s nutritional knowledge in a negative way. The reason for this result might be that children are confronted with high amounts of unhealthy food placements [8] and additionally, a lot of misleading health claims within media presentations [1,3]. The more children are exposed to such misleading claims over time, the less correct nutritional knowledge they develop. Thus, we can conclude that media consumption is a key driver for the development of misinformed food consumers.

Second, we observed that active or restrictive parental food-related mediation styles did not show any direct effects on children’s nutritional knowledge over time. Especially with respect to active mediation, this is a surprising finding. One explanation may be that parents may not necessarily teach detailed aspects of food and nutrition when they talk to their children, such as the amount of salt, sugar, or fat. By contrast, they may generally explain that something is healthy or not healthy. Rephrased, children may learn that a certain kind of food is good or bad for them, but that does not necessarily mean they can correctly estimate the amount of sugar, fat, or salt. This may be especially true considering the age range of the children who participated in our study.

Additionally, there were no interaction effects of active parental food mediation and children’s amount of media consumption. This again highlights the problems of parental mediation to lever out the effects of media content [11,37]. That is, even when parents talk actively about foods, this does not prevent the media negatively impacting nutritional knowledge. We believe there are at least two reasons for this: First, children are more frequently exposed to misleading health claims in the media [1,3] than they are exposed to parents’ explanations about the nutritional values of foods. In other words, given the high amounts of media consumption [33], children may see misleading health claims almost every day, but do not talk about this topic with their parents every day. Second, besides frequency, one may argue that misleading health claims are particularly persuasive [8]. That is, foods low in nutritional value are portrayed as desirable and they are consumed and liked by positively evaluated characters. Children may transfer this positive evaluation to other positive food characteristics, such as nutritional aspects. In terms of persuasion theory, these effects can be described as affect-based [8]. Against this background, it comes as no surprise that cognitive strategies, such as parents’ explanations of food characteristics, cannot beat affect-based effects generated by exposure to entertaining media content.

Third, we found an interaction effect of restrictive parental food-related mediation and children’s audiovisual media consumption on nutritional knowledge: The negative effect of media consumption on children’s nutritional knowledge over time increased with rising levels of restrictive mediation. This is striking because it indicates that if parents just enforce rules at home without giving sufficient argumentations [28], they leave their children solely subjected to the nutritional information provided in the media. Thus, a high amount of media consumption without parental food-related information actually leads to an accumulation of misinformation that negatively affects children’s understanding of nutrition.

In addition to these key insights, we found that the children’s age was a positive predictor for nutritional knowledge. However, BMI, and sex did not show any influence on children’s nutritional knowledge over time.

*Practical Implications.* Concluding from these results, reducing the amount of media consumption seems to be the most effective way to prevent a negative development of children’s nutritional knowledge. Additionally, the content of the media that children are exposed to needs to be carefully examined. Although, overall, there is a clear misrepresentation of nutritional facts in media targeted at children, they may also be shows and programs that deliberatively provide correct and realistic depictions of foods. Ultimately, it is the content that matters [8], not just exposure, and even though actively mediating about food does not lever out the negative effects on nutritional knowledge [11,37], active mediation about food may have other positive effects for the food habits of children. In other words, active mediation would not do any harm. Finally, we recommend parents to refrain from restrictive food-related mediation. Providing rules without explanations [28] leaves children fully subjected to the misinformation prevalent in media presentations [1,3].

*Limitations and Future Research.* As with every study, our study also faces some limitations. First of all, and most importantly, even though we assessed media as an agent for knowledge, we did not look into the actual content. While content analyses clearly state that misinformation about food in media presentations is very common [1,3], future research should combine content analytical data with survey data using linkage analysis [38]. Overall, self-report measures as used in our study are prone to measurement error [39]. Thus, additional experimental research is needed.

Second, the parental food-related mediation styles are indicators of the amount of information that parents provide to their children. That is, parents provide more information about foods when they engage in active food-related mediation as compared to restrictive food-related mediation [29]. This, however, does not assess the quality of information that children are provided with by their parents. Here, future research should assess the nutritional knowledge of parents.

Third, we tested nutritional knowledge based on the knowledge about the amounts of fat, sugar, and salt. Although this is an established and valid measure [35], and children also base their evaluation of what is healthy or not on these ingredients [21], there are other ways to test nutritional knowledge. In addition, we have employed the measure put forth by Tarabashkina and colleagues [36] for a slightly younger age-range than it has been intended for. Our study includes children starting at the age of five, while Tarabashkina and colleagues have validated their measure for children starting at the age of seven. Age is an important factor to consider when conducting research with children. For instance, especially for older children (i.e., above eleven years), knowledge can be measured with meal preparation tasks, which highlight the applicability of nutritional knowledge [18]. However, meal preparation tasks are difficult to measure in a panel context. Nevertheless, it seems important to replicate the results of our study with alternative nutritional knowledge measurements.

Fourth, we used a time-frame of six months to observe over time effects. Still, longer time-frames and more panel waves are recommended as a fruitful future research endeavor.

## 5. Conclusions

Given that movies and TV shows typically present an unhealthy food environment to children [8], full of misinformation [1,3], we demonstrated in an externally valid panel design with a large timespan that the amount of media consumption decreases nutritional knowledge in children over time. This is an alarming finding, suggesting that the current media environment produces young, misinformed food consumers [18]. What is even more alarming, our findings suggest that parents have only limited potential to counteract the effects of media consumption. Additionally, using a restrictive food-related mediation style could even strengthen the negative effects of media consumption on children’s nutritional knowledge over time. Therefore, we recommend that parents should attempt to reduce children’s amount of media consumption to develop and sustain nutritional knowledge in children.

## Figures and Tables

**Table 1:** Results for regression analysis predicting Time 2 of children’s nutritional knowledge.

Full Model: Main & Interaction Effects.	β	*SE*	*p*-Value
*Step 1: Individual Preconditions*			
Child Age	0.18	0.03	<0.001
Child BMIz ^a^	−0.01	0.03	0.726
Child Sex	0.01	0.06	0.870
Nutritional Knowledge (*t*1)	0.32	0.04	<0.001
*Step 2: Agents of Knowledge*			
Amount of Media Consumption	−0.11	0.05	0.003
Active Food-Related Mediation	0.05	0.02	0.232
Restrictive Food-Related Mediation	−0.02	0.02	0.694
*Step 3: Interaction Effects*			
Amount of Media Consumption × Active Food-Related Mediation	−0.01	0.04	0.866
Amount of Media Consumption × Restrictive Food-Related Mediation	−0.10	0.03	0.017
*R* ^2^	18.40%		

**Note**. *N* = 658 (Missing data *N_missings_* = 34). ^a^ Child BMIz is the standard deviation score of BMI to adjust for children’s sex and age.

## Appendix A

### Appendix A.1. Nutritional Knowledge Test Items: Wave 1

**Item 1: Which One Has the Lowest Amount of Fat?**					
Cookie	Cucumber (*correct answer*)	French fries	Coconut	Cheese	Don’t know

**Item 2: Which One Has the Highest Amount of Sugar?**					
Fruit juice	Banana	Cola (*correct answer*)	Muffin	Apple	Don’t know

**Item 3: Which One Has the Lowest Amount of Salt?**					
Potato chips	Bread	Cucumber (*correct answer*)	2 Oreo cookies	French fries	Don’t know

### Appendix A.2. Nutritional Knowledge Test Items: Wave 2

**Item 1: Which One Has the Lowest Amount of Fat?**					
Coconut	Yoghurt with fruits	Orange (*correct answer*)	Granola bar	Pizza	Don’t know

**Item 2: Which One Has the Highest Amount of Sugar?**					
Fanta (*correct answer*)	Grapes	Cake	Coconut	Mineral with raspberry flavor	Don’t know

**Item 3: Which One Has the Lowest Amount of Salt?**					
Cake	Pizza	Popcorn	Orange (*correct answer*)	Bread	Don’t know

### Appendix A.3. Measures Parental Food-Related Mediation

*Restrictive food-related Mediation*	I follow clear rules and restrictions on what my child is allowed to eat. I tell my child how much to eat of certain foods. I forbid my child to eat certain foods.
*Active food-related Mediation*	I talk to my child about why a food is healthy or unhealthy. I talk to my child about the consequences of unhealthy eating behavior. I instruct my child during the meal on which foods are healthy.

## References

[1] Roberts M., Pettigrew S. (2007). A thematic content analysis of children’s food advertising. Int. J. Advert..

[2] Jenkin G., Madhvani N., Signal L., Bowers S. (2014). A systematic review of persuasive marketing techniques to promote food to children on television: Persuasive TV food marketing to children. Obes. Rev..

[3] Castonguay J., McKinley C., Kunkel D. (2013). Health-related messages in food advertisements targeting children. Health Educ..

[4] Kelly B., King L., Baur L., Rayner M., Lobstein T., Monteiro C., Macmullan J., Mohan S., Barquera S., Friel S. (2013). Monitoring food and non-alcoholic beverage promotions to children: Monitoring food promotions to children. Obes. Rev..

[5] Keller S.K., Schulz P.J. (2011). Distorted food pyramid in kids programmes: A content analysis of television advertising watched in Switzerland. Eur. J. Public Health.

[6] Araque-Padilla R., Villegas-Navas V., Montero-Simo M.-J. (2019). Non-branded food placements in children’s entertainment programs: A content analysis. Health Commun..

[7] Speers S.E., Harris J.L., Schwartz M.B. (2011). Child and adolescent exposure to food and beverage brand appearances during Prime-Time television programming. Am. J. Prev. Med..

[8] Matthes J., Naderer B. (2019). Sugary, fatty, and prominent: Food and beverage appearances in children’s movies from 1991 to 2015. Pediatric Obes..

[9] Halford J.C.G., Gillespie J., Brown V., Pontin E.E., Dovey T.M. (2004). Effect of television advertisements for foods on food consumption in children. Appetite.

[10] Charry K.M. (2014). Product placement and the promotion of healthy food to pre-adolescents: When popular TV series make carrots look cool. Int. J. Advert..

[11] Naderer B., Matthes J., Binder A., Marquart F., Mayrhofer M., Obereder A., Spielvogel I. (2018). Shaping children’s healthy eating habits with food placements? Food placements of high and low nutritional value in cartoons, Children’s BMI, food-related parental mediation strategies, and food choice. Appetite.

[12] Fuller-Tyszkiewicz M., Skouteris H., Hardy L.L., Halse C. (2012). The associations between TV viewing, food intake, and BMI. A prospective analysis of data from the Longitudinal Study of Australian Children. Appetite.

[13] World Health Organization (2016). WHO Obesity and Overweight. Factsheet June 2016. http://www.who.int/mediacentre/factsheets/fs311/en/.

[14] Phipps S., Burton P., Lethbridge L., Osberg L. (2004). Measuring Obesity in Young Children. Can. Public Policy/Anal. De Polit..

[15] Shunk J.A., Birch L.L. (2004). Girls at risk for overweight at age 5 are at risk for dietary restraint, disinhibited overeating, weight concerns, and greater weight gain from 5 to 9 years. J. Am. Diet. Assoc..

[16] Rathi N., Riddell L., Worsley A. (2019). Parents’ and teachers’ critique of nutrition education in Indian secondary schools. Health Educ..

[17] Sherman J.W., Gawronski B., Gonsalkorale K., Hugenberg K., Allen T.J., Groom C.J. (2008). The self-regulation of automatic associations and behavioral impulses. Psychol. Rev..

[18] Harrison K. (2005). Is “Fat Free” good for me? A panel study of television viewing and children’s nutritional knowledge and reasoning. Health Commun..

[19] Worsley A. (2002). Nutrition knowledge and food consumption: Can nutrition knowledge change food behaviour?. Asia Pac. J. Clin. Nutr..

[20] Rathi N., Riddell L., Worsley A. (2017). Food and nutrition education in private Indian secondary schools. Health Educ..

[21] Elliott C., Brierley M. (2012). Healthy Choice?: Exploring How Children Evaluate the Healthfulness of Packaged Foods. Can. J. Public Health.

[22] Choi H., Paek H.-J., Whitehill King K. (2012). Are nutrient-content claims always effective?: Match-up effects between product type and claim type in food advertising. Int. J. Advert..

[23] Dixon H., Scully M., Niven P., Kelly B., Chapman K., Donovan R., Martin J., Baur L.A., Crawford D., Wakefield M. (2014). Effects of nutrient content claims, sports celebrity endorsements and premium offers on pre-adolescent children’s food preferences: Experimental research: Child responses to food pack promotions. Pediatric Obes..

[24] Harris J.L., Haraghey K.S., Lodolce M., Semenza N.L. (2018). Teaching children about good health? Halo effects in child-directed advertisements for unhealthy food: Halo effects in children’s food advertising. Pediatric Obes..

[25] Soldavini J., Crawford P., Ritchie L.D. (2012). Nutrition Claims Influence Health Perceptions and Taste Preferences in Fourth- and Fifth-Grade Children. J. Nutr. Educ. Behav..

[26] Birch L.L., Fisher J.O., Grimm-Thomas K., Markey C.N., Sawyer R., Johnson S.L. (2001). Confirmatory factor analysis of the Child Feeding Questionnaire: A measure of parental attitudes, beliefs and practices about child feeding and obesity proneness. Appetite.

[27] Birch L.L., Marlin D.W. (1982). I don’t like it; I never tried it: Effects of exposure on two-year-old children’s food preferences. Appetite.

[28] Borzekowski D.L.G., Robinson T.N. (2001). The 30-Second Effect. J. Am. Diet. Assoc..

[29] Buijzen M., Valkenburg P.M. (2005). Parental mediation of undesired advertising effects. J. Broadcast. Electron. Media.

[30] Lwin M.O., Shin W., Yee A.Z.H., Wardoyo R.J. (2017). A Parental Health Education Model of Children’s Food Consumption: Influence on Children’s Attitudes, Intention, and Consumption of Healthy and Unhealthy Foods. J. Health Commun..

[31] Reinehr T., Kersting M., Chahda C., Andler W. (2003). Nutritional knowledge of obese compared to non obese children. Nutr. Res..

[32] Childhood Obesity Surveillance Initiative (COSI) (2017). Bericht Österreich 2017. https://www.sozialministerium.at/Themen/Gesundheit/Kinder--und-Jugendgesundheit/COSI.html.

[33] Feierabend S., Plankenhorn T., Rathgeb T. (2016). KIM-Studie 2016 Kindheit, Internet, Medien. Basisuntersuchung Zum Medien.

[34] De Decker A., Sioen I., Verbeken S., Braet C., Michels N., De Henauw S. (2016). Associations of reward sensitivity with food consumption, activity pattern, and BMI in children. Appetite.

[35] Tarabashkina L., Quester P., Crouch R. (2016). Exploring the moderating effect of children’s nutritional knowledge on the relationship between product evaluations and food choice. Soc. Sci. Med..

[36] Hayes A.F. (2013). Introduction to Mediation, Moderation, and Conditional Process Analysis: A Regression-Based Approach.

[37] Naderer B., Matthes J., Marquart F., Mayrhofer M. (2018). Children’s attitudinal and behavioral reactions to product placements: Investigating the role of placement frequency, placement integration, and parental mediation. Int. J. Advert..

[38] Matthes J. (2008). Need for Orientation as a Predictor of Agenda-Setting Effects: Causal Evidence from a Two-Wave Panel Study. Int. J. Public Opin. Res..

[39] Scharkow M. (2019). The Reliability and Temporal Stability of Self-reported Media Exposure: A Meta-analysis. Commun. Methods Meas..

